# Molecular Cloning and Expression Profiles of Thermosensitive TRP Genes in *Agasicles hygrophila*

**DOI:** 10.3390/insects11080531

**Published:** 2020-08-13

**Authors:** Dong Jia, Zhouyu Ji, Xiaofang Yuan, Bin Zhang, Yanhong Liu, Jun Hu, Yuanxin Wang, Xianchun Li, Ruiyan Ma

**Affiliations:** 1 College of Plant Protection, Shanxi Agricultural University, Taigu 030801, China; biodong@sxau.edu.cn (D.J.); jzhouyu@126.com (Z.J.); xiaofyuu@foxmail.com (X.Y.); liuyanhong1984@126.com (Y.L.); hujun.yx@163.com (J.H.); wangyuanx1992@163.com (Y.W.); 2 College of Horticulture, Shanxi Agricultural University, Taigu 030801, China; jztgzhangbin@163.com; 3 Department of Entomology and BIO5 Institute, University of Arizona, Tucson, AZ 85721, USA

**Keywords:** activation, *Alternanthera philoxeroides*, expression profile, heat tolerance, thermosensation

## Abstract

**Simple Summary:**

The increase of hot days with temperatures over 37 °C in southern China due to global warming has led to summer collapse of the alligator weed flea beetle, an introduced biological agent for the invasive alligator weed. To promote understanding of the beetle’s adaption/tolerance to hot temperatures, we obtained *TRPA1*, *Painless*, and *Pyrexia*, three thermosensitive transient receptor potential channel genes from the beetle, and analyzed their expression patterns across different developmental stages and hot temperatures. Their constitutive expressions were dramatically different from each other and stage-specific. As temperature increased, their expressions in eggs elevated to their peak levels at 30 or 37.5 °C, and then fell back to their preferred temperature levels at temperatures > their peak temperatures. These results imply that (1) they may have different and stage-specific roles in perceiving high temperatures/chemicals and mediating the corresponding responses; and (2) their expressions may be decoupled from their activation. These findings lay a foundation for further understanding of the summer collapse of the beetle.

**Abstract:**

Global warming has gradually reduced the control efficacy of *Agasicles hygrophila* against the invasive weed *Alternanthera philoxeroides*. To better understand the summer collapse of *A. hygrophila* populations, we cloned the cDNA sequences of the high temperature-sensing *TRPA1*, *Painless*, and *Pyrexia* from *A. hygrophila*, and analyzed their temporal expressions and the impacts of high temperatures on their expression in eggs, the most vulnerable stage of *A. hygrophila* to hot temperatures. All the three genes obtained had the signature domains of TRPA channels and were constitutively expressed in eggs, larvae (L1, L2, L3), pupae, and adults, but *AhPainless* had the highest expression, followed by *AhPyrexia*, and *AhTRPA1*. The lowest and highest expression stages were adult and pupae for *AhTRPA1*, egg and L3 for *AhPainless*, and pupae/adult and L2 for *AhPyrexia*. The expressions of *AhTRPA1*, *AhPainless*, and *AhPyrexia* remained low at the preferred temperature range of 25–28 °C, elevated to their peak levels at 37.5, 30, and 30 °C, respectively, then fell to their 25–28 °C levels (*AhTRPA1*, *AhPainless*) or a lower level (*AhPyrexia*) at one or more temperatures >30 or 37.5 °C. These results suggest that their temperature-sensing roles and importance may be different, stage-specific, and their expression may be decoupled from their activation.

## 1. Introduction

Temperature is the most important environmental factor affecting all aspects of insect life, such as growth, development, reproduction, behavior, and survival [1]. Insects must perceive the temperature autonomously to find the microenvironment most suitable for their growth and reproduction while avoiding hot or cold temperatures. Therefore, they have developed various temperature-sensing mechanisms during their evolution [2,3]. The transient receptor potential (TRP) channels associated with the organismal level of temperature sensation, known as “thermoTRP” channels [4], are among the most commonly used temperature-sensing mechanisms. As a family of cation channels located on the cell membrane of numerous animal cell types, TRP channels also mediate sensation of visual, auditory, tactile, olfactory, taste, mechanical cues [5,6,7,8,9]. ThermoTRP channels are thermally activated to trigger various temperature-dependent in vivo responses, such as thermosensation, thermotaxis, and regulation of cellular/tissue functions at physiological body temperature in mammals. For ectothermic insects, thermoTRP channels are essential for the insects to perceive changes in the ambient temperature and thus for their survival and behavioral responses [6].

Based on their homology and characteristics of structural domains, insect TRP channels are divided into seven subfamilies, i.e., TRPC (canonical), TRPA (ankyrin), TRPM (melastatin), TRPV (vanilloid), TRPN (no mechanoreceptor potential C, NOMPC), TRPP (polycystin), and TRPML (mucolipin) [6,10,11]. TRP channel genes have been detected in insects such as *Drosophila melanogaster*, *Bombyx mori*, *Tribolium castaneum*, *Apis mellifera*, *Anopheles gambiae*, and *Bactrocera dorsalis* [11,12,13]. The functions of insect TRP channels have been studied most thoroughly in *D. melanogaster* due to its simple neurons and facile genetic manipulation. In *D. melanogaster*, thermoTRPs related to temperature sensation mainly belong to three subfamilies: TRPA (TRPA1, Painless and Pyrexia), TRPC (TRP and transient receptor potential-like, TRPL), and TRPV (Inactive). *Drosophila* TRPC and TRPV subfamilies are conducive to sensing low temperatures below 17.5–18 °C and thus are associated with cold sensation [14,15], whereas *Drosophila* TRPA subfamily is related to heat sensation and plays a central role in high temperature detection and behavioral responses to high temperature [16].

TRP channels are cation ion permeable channels formed as a homo- or hetero-tetramer. Each TRP channel subunit encoding by a TRP gene is often composed of an intracellular N-terminal domain, 3–6 Ankyrin repeats, a six transmembrane α-helices domain, an ion pore-forming loop between transmembrane helices 5 and 6, and an intracellular C-terminal domain [8]. Each Ankyrin repeat is ≈33 residues in length and is structurally consisting of two α-helices connected by a β-turn [17]. TRPA subfamily is distinguished by having an unusually large number (>6) of Ankyrin repeats [17]. Ankyrin repeats occur in tandem arrangements to form elongated domains critical for many physiological processes [18].

There are three TRPA channels, including TRPA1, painless, and pyrexia, involved in detection and avoidance of high temperatures in *D. melanogaster*. TRPA1 is activated above 27 °C [19] to trigger thermotaxis from moderately elevated temperature of about 31–35 °C to a preferred temperature of 18–24 °C [20,21]. Recent studies have showed that the temperature activation threshold of TRPA1 is actually variable, depending on the TRPA1 isoform [22,23,24]. Pyrexia is activated above 35 °C to prevent *Drosophila* from high-temperature-induced paralysis [25,26]. Painless is opened at temperatures greater than 42 °C to initiate avoidance of hazardous temperature by *Drosophila* larvae and adults [27]. In other insects such as *T. castaneum*, TRPA1, painless, and pyrexia work in a similar manner to mediate sensation of different ranges of high temperatures, heat tolerance, and acclimation [28]. In *B. mori*, maternal TRPA1 is also activated at temperatures higher than 21 °C during the embryonic stage to stimulate the female adults to release diapause hormone into the progeny eggs, inducing egg diapause [29].

Although activation (channel opening) and transcription are two functional aspects of TRPA channels, the expressions of the above TRPA channels are underexplored. While both TRPA1 and painless are required for normal aversive behaviors to allyl isothiocyanate, a nociceptive allelochemical that *D. melanogaster* may encounter in its host plants, the two channels are expressed in different neurons of the peripheral (leg and labral sense organs) and central nervous systems [27,30]. Similarly, the spatiotemporal expression patterns of *TRPA1*, *painless*, and *pyrexia* are at least partially different in the oriental fruit fly (*Bactrocera dorsalis*) [13]. In rat where TRPA1 is activated by a wide range of environmental chemicals and decreases in temperature (at below 17 °C), cold temperature (five weeks at +4 ± 6 °C) does not affect its expression but inhibits the expression of TRPV3 that is activated at temperatures above 31 °C [31]. For comparison, the rat TRPA1 activator formaldehyde enhances mRNA and protein levels of TRPA1, whereas the rat TRPA1 blocker menthol reduces TRPA1 expression [32].

The alligator weed flea beetle (*Agasicles hygrophila* Selman and Vogt) has been introduced from Argentina to successfully control the globally invasive weed *Alternanthera philoxeroides* (Mart.) Griseb in the United States, Australia, New Zealand, Thailand, and China [33,34]. The control efficacy of the beetle against the weed has reduced dramatically in recent years due to the increase of hot days with 0.5–2 h maximum temperatures over 37 °C resulted from global warming in southern China [35]. While such hot summer permits the weed to proliferate rapidly, it suppresses the beetle population or even makes it collapse [36,37,38,39]. This is because the eggs of the beetle, the most vulnerable stage to heat stress, cannot tolerate temperatures over 37.5 °C for a short period of 1 h [40]. To promote understanding of the adaption/tolerance of the beetle population to a short period of hot temperatures, we cloned the cDNA sequences of *TRPA1*, *Painless*, and *Pyrexia*, the three TRPA genes involved in detection and avoidance of high temperatures in insects, from the beetle *A. hygrophila* and analyzed the expression profiles of the three TRPA genes and the impacts of 1 h of hot temperatures on their expression. We found that the constitutive expressions of the three genes were dramatically different from each other, stage-specific, and their expression fell back to their preferred temperature levels at temperatures > their peak temperatures. These results imply that (1) the three genes may have different and stage-specific roles in perceiving high temperatures/chemicals and mediating the corresponding behavioral responses; and (2) their expression may have nothing to do with their activation temperatures.

## 2. Materials and Methods

### 2.1. Insect and Host Plant

The *A. hygrophila* laboratory strain used in this study was established with a field collection from South China Agricultural University campus (Guangzhou, China) over 10 years ago. This laboratory strain has since been maintained on *A. philoxeroides* plants, which was initially collected from Yuhuan County, Zhejiang Province over 10 years ago, and has since been planted in greenhouses of Shanxi Agricultural University, in the insectary of Shanxi Agricultural University (Taigu, Shanxi, China) under standard conditions (25 ± 1 °C, light:dark = 14:10 h, relative humidity (RH) = 85% ± 5%) [40].

### 2.2. Heat Shock of Beetle Eggs and Collection of Beetles of Different Stages

*A. philoxeroides* plants with *A. hygrophila* eggs laid within 3 h (8–11 am) at 25 °C were exposed to 25 °C (control), 27.5, 30, 32.5, 35, 37.5, 40, or 42.5 (±0.5 °C) at 85% ± 5% RH, which was equal to the average RH in the summer of southern China, the habitat of the alligator weed beetle, for 1 h [40]. The eggs were then gently detached from the weed leaves, flash-frozen with liquid nitrogen, and stored at −80 °C for subsequent total RNA extraction. Three replicates of 20 egg masses (25–30 eggs per mass) each were performed for control and each temperature treatment. To ensure that the fluctuation of the tested temperature was ≤±0.5 °C, the growth chambers with treated eggs were monitored and corrected in real time using HOBO U12 Temp/RH/Light/External Data Logger (Onset Computer Corporation, Bourne, MA, USA).

We also randomly collected, flash-frozen with liquid nitrogen, and stored at −80 °C three replicates of beetles of different developmental stages from the laboratory strain for subsequent total RNA extraction. These included 1-day-old eggs (20 egg masses per replicate), 1-day-old 1st (100–120 larvae per replicate), 2nd (80–100 larvae per replicate), and 3rd (60–80 larvae per replicate) instar larvae, 2-day-old pupae (48 h after boring into plant stems; 20 pupae per replicate), and 1-day-old adults (six males + six females per replicate).

### 2.3. RNA Extraction

Total RNAs were extracted from the *A. hygrophila* samples of different developmental stages and the heat-shocked *A. hygrophila* egg samples prepared above using TRIzol reagent (Life Technologies, Carlsbad, CA, USA) according to the manufacturer’s protocol. The concentration and purity of the obtained RNA samples were measured using BioPhotometer Plus (Eppendorf, Hamburg, Germany), and the integrity of each RNA sample was verified using 1% agarose gel electrophoresis.

### 2.4. RT-PCR Cloning of Three TRPA Genes

The RNA sample used for RT-PCR cloning of the three *TRPA* genes was prepared by pooling an equal amount of RNA from each of the six developmental stages. A total of 1 μg of this pooled total RNA sample was reverse transcribed into cDNA using M-MLV Reverse Transcriptase (Promega, Madison, WI, USA). Then, 1 μL of the resultant cDNA sample was used as the template to RT-PCR-amplify the cDNA sequences of *TRPA1*, *painless*, and *pyrexia*, respectively, in a 25 μL reaction containing 12.5 μL 2 × Phanta Max Buffer, 1 μL Phanta Max Super-Fidelity DNA Polymerase (Vazyme Biotech, Nanjing, China), 6.5 μL ddH_2_O, and 2.0 μL of the gene-specific forward and reverse primers (10 μM) (Table S1) designed based on the contigs of each gene found in the full-length transcriptome of *A. hygrophila* [41]. The amplification conditions of PCR were pre-denaturation at 94 °C for 3 min, followed by 35 cycles of denaturation at 94 °C for 30 s, annealing at 56 °C for 30 s, and elongation at 72 °C for 4 min, as well as a final elongation at 72 °C for 5 min. The obtained RT-PCR products of each gene were fractioned on a 1.2% agarose gel, eluted using a MiniBEST Agarose Gel DNA Extraction Kit Ver.4.0 (TaKaRa, Dalian, China), and cloned into pMD™19-T Vector (TaKaRa, Dalian, China). Three positive clones for each gene were sequenced (Sangon Biotech Co., Ltd., Shanghai, China).

### 2.5. Sequence and Phylogenetic Analysis

The open reading frames (ORFs) and amino acid sequences of the three TRPA genes were predicted using NCBI ORF finder (https://www.ncbi.nlm.nih.gov/orffinder/). Their isoelectric points and molecular masses were predicted using the ExPASy program ProtParam (http://web.expasy.org/protparam/). Their potential transmembrane domains were predicted using TMHMM Server v.2.0 (http://www.cbs.dtu.dk/services/TMHMM/). Their conserved TRPA domains were confirmed using Pfam and SMART (http://smart.embl-heidelberg.de/) [42]. Amino acid sequence alignments of *A. hygrophila* TRPA1 (AhTRPA1), Painless (AhPainless), Pyrexia (AhPyrexia) with their homologues from insects of different orders were done with the ClustalW program in MEGA7.0. The neighbor-joining phylogenetic trees of the three TRPA proteins were constructed using the p-distance model in MEGA7.0 program using [43]. Node support was assessed using a bootstrap procedure based on 1000 replicates.

### 2.6. Reverse Transcription Quantitative PCR (RT-qPCR)

A total of 800 ng of each RNA sample were used as the template to synthesize the first-strand cDNA using a PrimeScript RT reagent Kit with gDNA Eraser (Perfect Real Time) (TaKaRa, Dalian, China). Reverse transcription quantitative PCR (RT-qPCR) analyses of the expression of the three *TRPA* genes were performed on an ABI 7500 (Applied Biosystems, Foster City, CA, USA) with SYBR Green Real-time PCR Master Mix (Toyobo, Osaka, Japan). Their gene-specific primers (Table S1) to amplify an 80–150-bp product were designed using online software primer3 (http://primer3.ut.ee). The PCR conditions were 95 °C for 2 min, followed by 40 cycles of 95 °C for 15 s, 60 °C for 15 s, and 72 °C for 30 s. After the final PCR cycle of each gene, melting curve was yielded to verify the absence of junk by measuring the fluorescence from 60 to 95 °C. The *RPS18* and *β-actin* genes were used as the internal reference genes for RT-qPCR analyses of the three *TRPA* genes in heat-shocked eggs [44], whereas the *RPS18* and *RPL13a* genes were used as the internal reference genes for RT-qPCR analyses of the three *TRPA* genes in beetles of different developmental stages [45].

For each target (i.e., *TRPA* genes here) and reference gene, we had three biological replicates (RNA samples) of three technical repeats each. The normalized expression level of each *TRPA* gene was calculated according to the following two equations [46].

(1) $$\mathrm{Expression}\;\mathrm{level}=(1+E_{\mathrm{gene}}{)}^{-Ct_{\mathrm{gene}}}$$

(2) $$\mathrm{Normalized}\;\mathrm{expression}\;\mathrm{level}\;\mathrm{of}\;\mathrm{target}\;\mathrm{gene}=\frac{{\left(1+E_{\mathrm{target}\;\mathrm{gene}}\right)}^{-Ct}{}^{{}_{\mathrm{target}\;\mathrm{gene}}}}{\sqrt[{2}]{{\left(1+E_{\mathrm{ref}\;\mathrm{gene}1}\right)}^{-Ct_{\mathrm{ref}\;\mathrm{gene}1}}\times {\left(1+E_{\mathrm{ref}\;\mathrm{gene}2}\right)}^{-Ct_{\mathrm{ref}\;\mathrm{gene}2}}}}$$

One-way ANOVA followed by Tukey’s honest significant difference (HSD) test at *p* < 0.05 was performed to determine if the normalized expression level of *AhTRPA1*, *AhPailess*, or *AhPyrexia* was significantly different among different developmental stages or different temperature treatments using SPSS 21.0 software (IBM, Armonk, NY, USA). All the histograms were drawn using SigmaPlot 12.5 (Systat Software Inc., San Jose, CA, USA).

## 3. Results

### 3.1. Cloning and Sequence Analysis of A. hygrophila TRPA Genes

**Figure 1:**
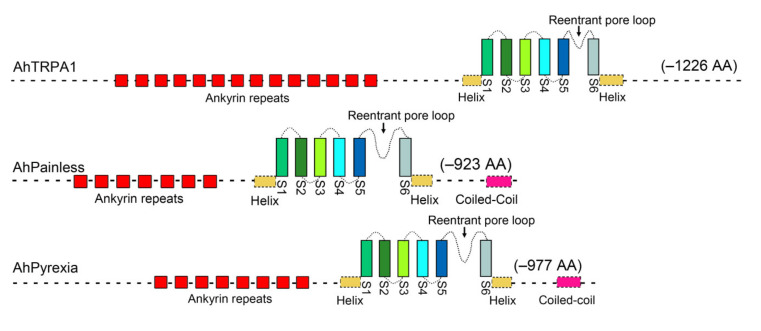
Predicted structural domains of TRPA channels. AhTRPA1, AhPainless, and AhPyrexia were three TRPA channels of *Agasicles hygrophila*, all of which have six transmembrane damains (S1–S6), and a reentrant pore loop between transmembrane domains S5 and S6.

The complete ORF of the *A. hygrophila TRPA1* (*AhTRPA1*) gene is 3681 bp in length and encodes a protein of 1226 amino acids, with a molecular mass of 138.90 kDa and an isoelectric point of 7.42. It contains 14 ankyrin repeats from amino acid (AA) 151–666 at the N-terminus and six ion channel-forming transmembrane domains from AA 807–1063 at the C-terminus (Figure 1), (Figure S1). The complete ORF of *AhPainless* gene is 2772 bp in length and encodes a protein of 923 amino acids, with a molecular mass of 106.15 kDa and an isoelectric point of 7.45. AhPainless has seven ankyrin repeats located in AA 62–420 at the N-terminus, and six ion channel-forming transmembrane domains located in AA 532–772, and a coiled coil in AA 891–923 at the C-terminus (Figure 1), (Figure S2). The complete ORF of the *AhPyrexia* gene is 2934 bp in length and encodes a protein of 977 amino acids, with a molecular mass of 110.10 kDa and an isoelectric point of 6.21. AhPyrexia contains eight Ankyrin repeats located in 190–459 at N-terminus, six ion channel-forming transmembrane domains located in in AA 554–809, and a coiled coil in AA 927–967 at the C-terminus (Figure 1), (Figure S3).

### 3.2. Phylogenetic Analysis of A. hygrophila TRPAs with TRPs from Other Insects

**Figure 2:**
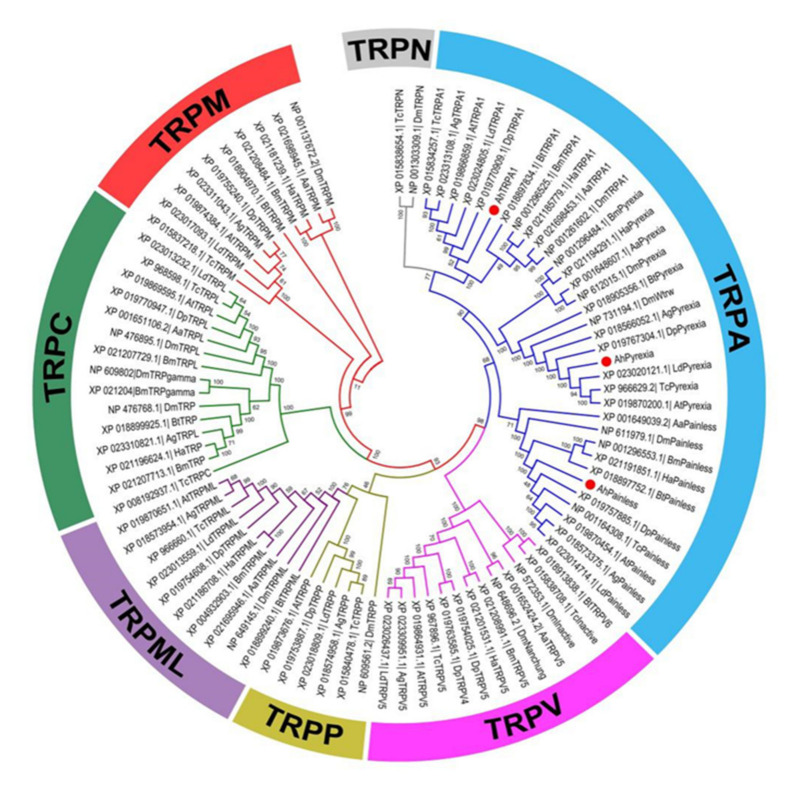
Phylogenetic analysis of transient receptor potentials (TRPs) in *Agasicles hygrophila* and other insects. Ninety TRP sequences from 11 species of four insect orders were used to construct this phylogenetic tree. The GenBank accession numbers of these sequences are listed in Table S2. The TRPA1, Painless, and Pyrexia of *Agasicles hygrophila* are represented by red dot “●”.

Phylogenetic analysis of AhTRPA1, AhPainless, and AhPyrexia with 87 TRPs from 10 species of four insect orders (Coleoptera, Diptera, Hemiptera, and Lepidoptera) placed the 90 insect TRPs in seven TRP subfamilies including TRPM, TRPC, TRPP, TRPML, TRPV, TRPA, and TRPN (Figure 2). Consistent with their structural characteristics, AhTRPA1, AhPainless, and AhPyrexia were clustered with other insect TRPA1, Painless and Pyrexia in the TRPA subfamily, respectively. Within the TRPA1, Painless, and Pyrexia branches, AhTRPA1, AhPyrexia, and AhPainless formed a smaller branch with TRPA1, Painless, and Pyrexia from other Coleopteran insects, respectively (Figure 2).

### 3.3. Temporal Expression Patterns of AhTRPA1, AhPainless, and AhPyrexia

**Figure 3:**
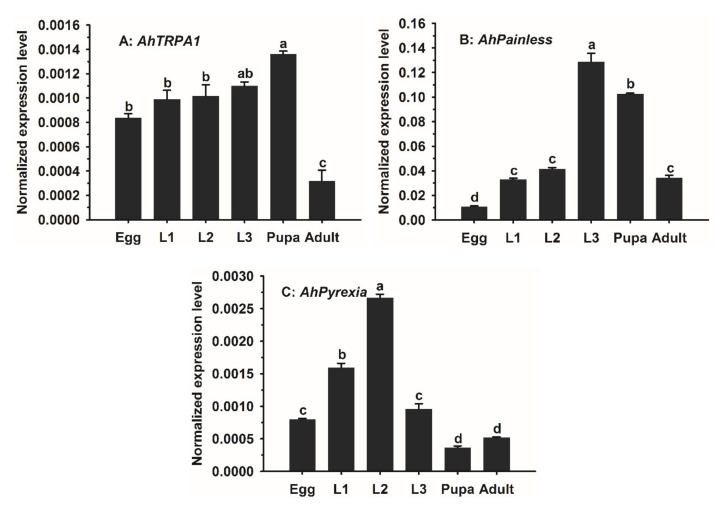
Developmental expression profiles of (**A**) *AhTRPA1*, (**B**) *AhPainless*, and (**C**) *AhPyrexia* in *Agasicles hygrophila*. Each developmental stage comprised three biological replicates. Data of the bar chart are expressed as mean ±SE. The differences in the expression during different developmental stages were analyzed using one-way ANOVA and Turkey’s honest significant difference (HSD) test. Different lowercase letters represent significant differences at *p* < 0.05.

The basal expression levels of the three genes during different developmental stages were analyzed by RT-qPCR. The results showed that the three *A. hygrophila TRPA* genes were constitutively expressed in all developmental stages, but their expression levels differed dramatically (Figure 3). *AhPainless* had the highest expression, followed by *AhPyrexia*, and *AhTRPA1*. Overall, the expression of *AhTRPA1* increased as the life stage advanced from egg to pupa and peaked in the pupal stage. However, the differences in the expression of *AhTRPA1* were not significant between egg and different larval stages (*p* > 0.05), and the lowest expression of *AhTRPA1* was observed in the adult stage (Figure 3A).

The expression level of *AhPainless* increased gradually from the egg to the third-instar larval stage (L3), and then gradually decreased from the third-instar larval stage to pupal and adult stages (Figure 3B). Multiple comparison showed that the third-instar larvae had the highest level of *AhPainless*, followed by pupae, then L1, L2 and adults, and finally eggs. The expression levels of L3, pupae, and adults were 11.8, 9.4, and 3.1 times that of eggs, respectively (Figure 3B).

The expression level of *AhPyrexia* was the highest in L2, followed by L1, egg and L3, and finally pupae and adults (Figure 3C). The expression of *AhPyrexia* in L2 was significantly higher than in L1. No significant difference in *AhPyrexia* expression was found between egg and L3 as well as between pupae and adults. L1 had significantly greater expression of *AhPyrexia* than did egg and L3. Both egg and L3 exhibited significantly higher expression of *AhPyrexia* than pupae and adults (Figure 3C).

### 3.4. Impacts of Heat Shock on the Expression of AhTRPA1, Ahpainless, and AhPyrexia

**Figure 4:**
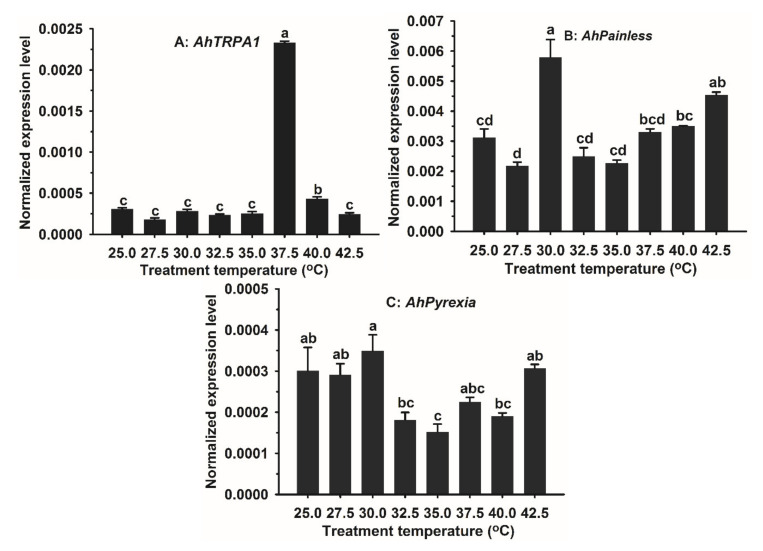
Impacts of temperature on the expression of (**A**) *AhTRPA1*, (**B**) *AhPainless*, and (**C**) *AhPyrexia* in *Agasicles hygrophila* eggs. Each treatment included three biological replicates. Data of the bar chart are represented by mean ±SE. Differences in expression at different high temperatures were analyzed using one-way ANOVA and Turkey’s HSD test. Different lowercase letters represent significant difference at *p* < 0.05.

As egg was the most vulnerable stage of *A. hygrophila* to hot temperature in the summer [40], we exposed its eggs to 25 (control), 27.5, 30, 32.5, 35, 37.5, 40, or 42.5 °C for 1 h and then analyzed the expressions of *AhTRPA1*, *AhPainless*, and *AhPyrexia* genes in the *A. hygrophila* eggs treated with one of the eight different temperatures. The expression of *AhTRPA1* remained unchanged from 25–35 °C, sharply elevated 7.3-fold to its highest level (peak expression) at 37.5 °C, dramatically reduced 6.0-fold at 40 °C, and finally dropped back to the expression level of 25–35 °C at 42.5 °C (Figure 4A). The expression of *AhPainless* did not change significantly with the increase of temperature except for 30 and 42.5 °C that significantly induced expression of this gene (Figure 4B). The peak expression of *AhPainless* occurred at 30 °C, which was marginally higher than that at 42.5 °C (Figure 4B). Similarly, the expression of *AhPyrexia* did not change significantly with the increase of temperature except for 35 °C (Figure 4C). The expression of *AhPyrexia* at 35 °C was significantly lower than that at 25, 27.5, 30, and 42.5 °C, respectively (Figure 4C).

## 4. Discussion

In this study, the “thermoTRP” channels TRPA1, Painless, and Pyrexia known to be activated by temperatures higher than the optimal temperature (18–24 °C) of *D. melanogaster* [16] and noncaptive allelochemicals [30] were cloned from the alligator weed flea beetle *A. hygrophila* that prefers living at 25–28 °C (Jia et al., personal observation) but often encounters hot temperature in the summer season of southern China [40]. The encoded protein sequences of the *AhTRPA1*, *AhPainless*, and *AhPyrexia* we obtained from *A. hygrophila* have 7–14 tandem ankyrin repeats (Anks) of about 33 amino acids each (Figure 1), (Figures S1–S3), a signature feature of the TRPA subfamily. The tandem repeat of multiple Anks may be the important component for these TRPA channels to sense and transmit temperature stimuli [4,17,18]. AhTRPA1, AhPainless, and AhPyrexia also have six highly conservative transmembrane helices (S1–S6) with a reentrant pore loop between the fifth and sixth transmembrane helices (Figure 1), the common structure feature of TRP channels [8,47]. These two features plus the finding that *AhTRPA1*, *AhPyrexia*, and *AhPainless* clustered with the TRPA1, Painless, and Pyrexia from other Coleopteran insects (Figure 2), respectively, confirm that the *AhTRPA1*, *AhPyrexia*, and *AhPainless* we cloned are the canonical *TRPA1*, *Painless*, and *Pyrexia* genes of *A. hygrophila*.

Analysis of the temporal expression patterns of *AhTRPA1*, *AhPainless*, and *AhPyrexia* reveals two common and two gene-specific features. One common feature of the three *A. hygrophila TRPA* genes is that they were all expressed at the six tested stages. This suggests they all play a role in sensing high temperatures/or chemicals and mediating the corresponding responses in every life stage of this beetle. Another common feature is that their expression levels varied significantly among different developmental stages. This indicates that they may have stage-specific functions and/or relative importance in sensing high temperatures/chemicals and mediating the corresponding responses. One of the two gene-specific features is that the lowest and highest expression stages were adult and pupae for *AhTRPA1*, egg and L3 larvae for *AhPainless*, and pupae/adult and L2 larvae for *AhPyrexia*. This led us to speculate that *AhTRPA1*, *AhPainless*, and *AhPyrexia* contribute the least and the most to thermosensory responses in adult and pupae, egg and L3 larvae, and pupae/adult and L2 larvae, respectively. Another gene-specific feature is that *AhPainless* had the highest expression, followed by *AhPyrexia*, and *AhTRPA1*. This suggests that *AhPainless* may play more important roles than *AhPyrexia*, and *AhTRPA1* may be the least important one of the three TRPA genes. Consistent with our findings, such unequal and stage-specific expression of the three genes have been detected in other insects [13,48,49].

While we do not know the exact temperature thresholds that activate/open AhTRPA1, AhPainless, and AhPyrexia, respectively, it is expected that the thresholds of the three TRPA channels should be higher than 25–28 °C, the preferred living temperature range of this beetle (Jia, Personal observation). The inhibition of the high temperature (>31 °C) activated rat *TRPV3* expression by cold temperature [31] as well as the inhibition and induction of rat *TRPA1* by its blocker menthol and activator formaldehyde [32] led us to hypothesize that the expression of the three *A. hygrophila TRPA* genes should remain at a constitutive level at 25–28 °C and increase to a higher level once the temperature reaches their corresponding thresholds. Analysis of the expressions of the three genes in *A. hygrophila* eggs treated with different temperatures from 25 to 42.5 °C, however, did not support this prediction. This is because the expressions of *AhTRPA1*, *AhPainless*, and *AhPyrexia* elevated to their peak expression levels at 37.5, 30, and 30 °C, respectively, but all fell to their 25 °C levels (*AhTRPA1*, *AhPyrexia*) or an even lower level (*AhPainless*) at one or more of the temperatures greater than 30 or 37.5 °C (Figure 3). Two possibilities may explain this discrepancy. First, the activation or opening of the three *A. hygrophila* TRPA channels, unlike rat TRP channels [31,32], has nothing to do with their expression levels. Second, the three channels may be closed again at the temperatures greater than their peak expression temperatures (*T*_peak_). Functional determination of their activation and closing temperatures by two-electrode voltage clamp assays of *Xenopus laevis* oocytes heterologously expressed with *AhTRPA1*, *AhPainless*, or *AhPyrexia* are required to resolve the two possibilities.

Interestingly, the *T*_peak_ of *AhTRPA1* in *A. hygrophila* eggs, 37.5 °C, is exactly the same with the high temperature tolerance limit of *A. hygrophila* egg, at and above which the egg hatching rate is significantly reduced [40], the major cause of the summer collapse of *A. hygrophila* populations in southern China [36,37,38,39]. While use of the *T*_peak_ of TRP genes has not been reported, the onset (*T*_on_) and peak (*T*_peak_) expression temperatures of heat shock proteins (HSPs) have been suggested as the useful indicators of the cold and hot temperature tolerance limits and thus northern and southern distribution boundaries of a given species [50]. Following this suggestion, *T*_on_ and *T*_peak_ of HSPs have been studied and applied as the biological indicators in evaluating thermal tolerance, diapause and geographical distribution of many species [51,52,53,54,55,56]. The fact that 21 out of 26 *A. hygrophila* HSP genes have a *T*_peak_ of 37.5 °C in eggs [44] proves the utility of the *T*_peak_ of HSP gene as the heat tolerance limit indicator. Whether the perfect match of the *T*_peak_ of *AhTRPA1* with the hot temperature tolerance limit of *A. hygrophila* egg found here is just a coincidence or a common phenomenon in insects necessitates a correlation analysis of *T*_peak_ of *TRPA1* and heat tolerance limits in a number of insect species.

## 5. Conclusions

In this study, three thermosensitive TRP genes, including *TRPA1*, *Painless*, and *Pyrexia* in *A. hygrophila* were characterized and proved to belong to the canonical TRPA subfamily by structure domain and phylogeny analyses. We found the three TRPA genes were all expressed at the six tested stages but differed significantly in the expression level. As temperature increased from the preferred living temperature range (25–28 °C) of *A. hygrophila*, the expressions of the three *TRPA* genes in eggs elevated to their peak levels at 30 or 37.5 °C, but then all fell back to their 25 °C or even lower levels. The *T*_peak_ for *AhTRPA1* in *A. hygrophila* eggs was the same with the high temperature tolerance limit of *A. hygrophila* eggs, but this could be just a coincidence.

## Supplementary Materials

The following are available online at https://www.mdpi.com/2075-4450/11/8/531/s1, Figure S1: Putative amino acid sequence of AhTRPA1. Numbers on the left indicate the locations of amino acid residues. Shaded areas represent the predicted ankyrin repeats (Ank1–Ank14). Framed sequences were the predicted transmembrane segments (S1–S6), Figure S2: Putative amino acid sequence of AhPainless. Numbers on the left indicate the locations of amino acid residues. Shaded areas represent the predicted ankyrin repeats (Ank1–Ank7). Framed sequences were the predicted transmembrane segments (S1–S6), Figure S3: Putative amino acid sequence of AhPyrexia. Numbers on the left indicate the locations of amino acid residues. Shaded areas represent the predicted ankyrin repeats (Ank1–Ank8). Framed sequences were the predicted transmembrane segments (S1–S6), Table S1: Primers used for RT-PCR cloning of the full-length cDNA sequences of *AhTRPA1*, *AhPailess*, *AhPyrexia* and qPCR analysis of their expression, Table S2: The accession numbers of all TRPs used in phylogenetic analysis.
