# Supplementary material for: Molecular Cloning and Expression Profiles of Thermosensitive TRP Genes in Agasicles hygrophila
Source: Insects. 2020 Aug 13;11(8):531. doi: 10.3390/insects11080531 (PMC7570112; doi:10.3390/insects11080531)
Supplement: Supplementary file 1 [file insects-11-00531-s001.pdf]

insects

Supplemental Material

Molecular cloning and expression profiles of thermosensitive TRP genes in *Agasicles hygrophila*

```
1    MDARMPRILQFLHALTTWRIDSENDYQQAPSTPIITIISP
41   VDEPVKNIGDNEATTEEGRMYGQPRRLPKIKDWIVNVFQI
81   FIKKIGFGSRKNKRDEQLSRTGSANEKPSDGLCHISQSPF
121  RILRAAESGNLEIFKRLYHEDISRLHIQDPRGRTPAHQAA
161  SRNKVNILQFILDQGGDLNAQDNCGNTPLHIAVEHAALDA
               Ank1                      Ank2
201  VDFLLSRKVKTDILNEKNQAPIHLVAELNKVSVLEVMGRY
                                 Ank3
241  KDKINIQQGGEHGRLALHIAAIYDHEECAKLLISEFGASP
                              Ank4
281  KIPCNNGYYPIHEAAKNASSKSLEVFLQWAESRGCSRQEM
                         Ank5
321  ISLYDSEGNVPLHSAVHGGDIRAVEICIKSGAKISIQQQD
                           Ank6
361  LSTPVHLACSQGATEIVKLMFTMQPEEKAACLCSCDIQKM
                Ank7
401  TPLHCAAMFDHPEIVEYLVLEGADINAMDKERRSPMLLAA
                 Ank8                   Ank9
441  SRGAWKTAQLLIRLDADVTITDINRRNLLHLVVMNGGRLE
481  RFTSEGKQKDTLLNLLNEKDIHGCSPLHYASREGHIRSLE
                                Ank10
521  TLINLGAVINLKNNNNESPLHFAARYGRYNTVKRLLDSDK
                           Ank11
561  GTFIINESDGGGLTPLHISSQQGHTKIVQLLLNRGALLHR
                          Ank12
601  DHTGRNPLHLAAMNGFTQTIELLLSVHSHLLDQTDKDGNT
                   Ank13
641  ALHVATIENQPRVISLLLTVRCKITNNHLDMSAIDYAIYY
               Ank14
681  KYCEAALAMVTHPERGDELLHYKSNKHRCITMALISSMPR
721  VFEAVQDKGITRADCKKDSISFYMKYSFSSLQGSILSDME
761  DDKNAKNWEPLPALNAMVLHGRVELLAHPLSTKYLQMKWN
801  SYGKYFHVANMTFYSIFLSFVTAFASQLMEQQIPTSPSNF
                       S1
841  TNMTHADYIQYTKQQILNIRISPAMYVSALGIFSYIGLNI
                                S2
881  VREIIQIWQQRYLYFMDPINLVTWLLYLSAFIMTLPIFGG
                         S3
921  DMHDLQFTCASLTVFLSWFNLLLLLQRFDQVGIYVVMFLE
                 S4
961  ILQTLIKVLMVFSILIIAFGLAFYILLSRGEHLSSEQYQC
                 S5
1001 PLVRTFSMMLGEIDFLGTYVKPYYMINGDEKSFLPFPLPA
1041 FFILGLFMVLMPILLMNLLIGLAVGDIESVRRNAQLKSLA
                S6
1081 MQVALHTELERKIPRFLLKRVDKMEVIEYPNETDGKGGIL
1121 EMILRKWFRTPFSDTSVGMPVEKTEDYILAELEKSKRKLR
1161 EISCCLEAQQQFLRLIVQKMEIKTEADDVDEGITNTTSPT
1201 EGSTKWNSPNIRKKVRSVVRFSNKQN
```

Figure S1. Putative amino acid sequence of AhTRPA1. Numbers on the left indicate the locations of amino acid residues. Shaded areas represent the predicted ankyrin repeats (Ank1–Ank14). Framed sequences were the predicted transmembrane segments (S1-S6).

1 MDTFKSTKYSKMDGPQSGAVVRTNSICPSPEDTLLQQVLQ

41 NDIDGIAKTINDNLINHIYEDYNKPILLIACMKNEVNEKT

Ank1

81 IAKILEYKPNLRYFDKEQWEALHYASSKTDSQVLKLVIDS

Ank2

121 IQKKYDINKILAQGNNALHILIKYGNLESDDFIKCAEILI

Ank3

161 QAGINVNLGDNKNYSPILLAAFKGLKPLVELIINNIQQVD

Ank4

201 LDSHTILGKSARDYIREKVLYNGTLPRKLNNNNQDHLLLV

241 NGNTEREIDILFTYIKKRDENGFVNFKNGNIENLLTCKGY

281 DNTLLQLACDVGTTKIVQHLIDNKADRLETTAKNKKTPIE

Ank5

321 IAADHGFFTIFKILLVDYNHILPTSVLINLIRYYNYETFE

Ank6

361 DIDRKKCCNHLLDEIDKKKVDIDINGYDDFDKNTPLHFAV

401 RYADADIIERLLKNGASLGSKNVCNIMPIRDIEPDLLEKH

Ank7

441 LDDCVQFNLKAKKEREEFEIDFDYRTLMAPYPVVEKKSAD

481 EESGKKQYQKQKMVHETEVISYMSGAPEFKHLMMHPVIAS

521 FLNMKWHRIQGLYFLNLGCYFAYCLFLVSYIFAYYANFDP

S1

561 VDSSQFSGFYNFLRYTSWVGLLITFIIIMFREIFQICIIP

S2

601 SKYFTQFENYVEFSLIILSGCILFIKSPSVQTRKQLSSVS

S3

641 ILFAAFELVLMLGQHPKFSTNVVMLRTVSYNFFKFFSWYC

S4

681 LLLIAFALSFHILFSEVPSVSASNGTDNVEEDDNFGNAGK

S5

721 SVFKTIIMLTGEFDASDLNFKPFPVVSKIIFVLFIFMIAI

S6

761 ILLNLLNGLAVSDTQMIKNDAEQIGHIYRAQHIYYVELMI

801 LGNVIPKYILKKINQLCCCLNINKDKYYSLFKPFTNKVCL

841 FNSPECKITMYPNRNGKLCISGDRKQSSCCSGWCNSHVEK

881 AIIRRTNEIVKARQNDKAEIEVNIKLLEKILKQLNSSVKK

921 SMD

Figure S2. Putative amino acid sequence of AhPainless. Numbers on the left indicate the locations of amino acid residues. Shaded areas represent the predicted ankyrin repeats (Ank1–Ank7). Framed sequences were the predicted transmembrane segments (S1-S6).

```
  1 M V S A S E N H S S F M M D S Y A F Q G S N G T T K R L G R S V S V N D E N T N
 41 F Q R Y R T P T S S T I L R W R K N R D D P E N I F E E E L P S D D F E Y M E C
 81 G P S P P A D H T P N I Y E S F E E F S P S A D L S V Q I C H D T I R L N L L E
121 H M K I S S G R H Q L L D D I E S K K I T L G G L T E C F Q G A S K A E V N I G
161 F L W S A F M K R H D L L E G F I K L G A Q L D Y Y E P S Q G L S A I H L T S F
                                                                Ank1
201 S G D N D G T Q L L I S K G C D V N A I Y K C Y T P L H C A A F G D S P E C A M
                                                            Ank2
241 I L L N N D A K V H I T T K S P Q N C R E S V L H C A V R A N A I A C V K L F T
                                                          Ank3
281 E E G A D V A Q G E N T G V T P I H L A A D L G H P Q C L R I L L E H K S I F V
                                                     Ank4
321 N T P T K D K E Q T P L H L A A E S G Y V E C I E I L L D N G A D A N V R N Y R
                                               Ank5
361 A Q S P L H L A A R A Q A Y D C V E M L L R K A N A D P K M Y D C D K R T P L H
                                       Ank6
401 C A V G K A A R S Y D I I E I L I S Y E A D I N A K D Q Y G Y T P L H I A A L N
                                  Ank7
441 E L S Q C V E V L L Y H G A D V T A K S K F G M T A L G I I T R K T P A S L A M
                             Ank8
481 V T Q K L D S A I T L H H H P E S S N R E V E L R L D F R G I L Q Y C H P R E I
521 S F L N T F V D E G Q K E I L L H P L C S A F L Y L K W E K I R K Y Y I A R M L
561 F C F I F V L S L S L Y V L T A L A H N C H N H G K N M N D T Q P Q N V I E L C
                            S1
601 E K K S M M G H M L R N N P F V I E M Q W F V L V A V T C C E I L R K V Y G I A
                                                       S2
641 G Y P S V R Q Y L S H P E N I I E W T V I A S V F V I S F I Y T G R T Y T W Q N
                                                  S3
681 H V G A I A V L F G W T N L M L M I G Q L P I F G S Y V A M Y T R V Q G E F A K
                                          S4
721 L L L A Y S C L L I G F T I S F C V M F P D S S T F A N P F I G L I T V M V M M
                                 S5
761 T G E L S L D L L V D D D P E D P P F L L E V S A Q V T Y I L F L L F V T V I L
                                                             S6
801 M N L L V G I A V H D I Q G L Q K T A G L S K L V R Q T K L I S Y M E L A L F N
841 G Y L P Q Y L L N L L H W T A L V S P K A Y R V V L N V K P L N P R E K R L P K
881 D I L K A A H E I A K M R K N Y G H T I S S Q T T T Y T K K L N N N L D Q N S D
921 I L D F S T F S L L Q G K I E K S S E Q I E H L S K E I R Q L K N T I E T N Q K
961 L L E Q L M T T I L H N T K N S K
```

Figure S3. Putative amino acid sequence of AhPyrexia. Numbers on the left indicate the locations of amino acid residues. Shaded areas represent the predicted ankyrin repeats (Ank1–Ank8). Framed sequences were the predicted transmembrane segments (S1-S6).

Table S1. Primers used for RT-PCR cloning of the full-length cDNA sequences of *AhTRPA1*, *AhPailess*, *AhPyrexia* and qPCR analysis of their expression.

**Purposes**	**Primer Names**	**Sequences (5'–3')**
qPCR	*AhTRPA1*-F:	AGGGGACATGCACGACTTAC
	AhTRPA1-R:	TTCCCACCTGATCAAACCTC
	AhPainless-F:	TGCAGACCCGTAAACAGTTG
	AhPainless-R:	CGAAACTGTTCTAAGCATCACG
	AhPyrexia-F:	TGTCAAGAGAGCGTGCTACA
	AhPyrexia-R:	TTGCGGGTGTCCTAGATCAG
	AhRPS18-F:	ACAAAATCCCCGACTGGTTC
	AhRPS18-R:	ATGGGCACGGATCTTCTTCA
	Ahβ-actin-F:	ACGAGGGTTATGCACTTCCA
	Ahβ-actin-R:	TGGTGAAAGAGTAGCCACGT
	AhRPL13a-F:	GAAGCGAGTAGTTGTGCCTG
	AhRPL13a-R:	TGCGAACGACTGTCTGGTAT
RT-PCR cloning	*AhTRPA1*-F:	AAATGTTAGTTTTTGTGCGGGAT
	AhTRPA1-R:	TACTTGTTGCTTTGTTGTGTGTT
	AhPainless-F:	CGATTACCAGTCCTGCAACG
	AhPainless-R:	TGCAGCATTTCTCTTCAGCA
	AhPyrexia-F:	TCAAACTTGGCGCTCAACTC
	AhPyrexia-R:	TGGAACCAATAATTGCGGCT

F: forward primer; R: reverse primer.

Table S2. The accession numbers of all TRPs used in phylogenetic analysis.

Categories	Species	Gene Names	Accession Numbers
TPRs	***Agasicles hygrophila***	*AhTRPA1*	MN540453
		AhPainless	MN540455
		AhPyrexia	MN540454
	Bemisia tabaci	*BtTRPA1*	XP_018897834.1
		BtPainless	XP_018897752.1
		BtPyrexia	XP_018905356.1
		BtTRPM	XP_018904970.1
		BtTRPV6	XP_018913838.1
		BtTRPML	XP_018899240.1
		BtTRP	XP_018899925.1
	Bombyx mori	*BmTRPA1*	NP_001296525.1
		BmPainless	NP_001296553.1
		BmPyrexia	NP_001296484.1
		BmTRPM	XP_021208484.1
		BmTRP	XP_021207713.1
		BmTRPL	XP_021207729.1
		BmTRPV5	XP_021208991.1
		BmTRPgamma	XP_021204805.1
		BmTRPML	XP_004932903.1
	Helicoverpa armigera	*HaTRPA1*	XP_021185779.1
		HaPainless	XP_021191851.1
		HaPyrexia	XP_021194291.1
		HaTRPV5	XP_021201531.1
		HaTRPM	XP_021181239.1
		HaTRP	XP_021196624.1
		HaTRPML	XP_021186708.1
	Tribolium castaneum	*TcTRPA1*	XP_015834257.1
		TcPainless	NP_001164308.1
		TcPyrexia	XP_966629.2
		TcTRPC	XP_008192937.1
		TcTRPM	XP_015837218.1
		TcTRPV5	XP_967896.1
		TcTRPML	XP_966660.1
		TcInactive	XP_015838708.1
		TcTRPL	XP_968598.1
		TcTRPP	XP_015840478.1
		TcTRPN	XP_015838654.1
	Anoplophora glabripennis	*AgTRPA1*	XP_023313108.1
		AgPainless	XP_018573375.1
		AgPyrexia	XP_018566052.1
		AgTRPM	XP_023311043.1
		AgTRPV5	XP_023309951.1
		AgTRPP	XP_018574958.1
		AgTRPML	XP_018573954.1
		AgTRPL	XP_023310821.1
	Leptinotarsa decemlineata	*LdTRPA1*	XP_023024805.1
		LdPainless	XP_023014714.1

	LdPyrexia	XP_023020121.1
	LdTRPM	XP_023017093.1
	LdTRPV5	XP_023026437.1
	LdTRPML	XP_023013559.1
	LdTRPL	XP_023013232.1
	LdTRPP	XP_023018809.1
Aethina tumida	*AtTRPA1*	XP_019866859.1
	AtPainless	XP_019870454.1
	AtPyrexia	XP_019870200.1
	AtTRPM	XP_019874384.1
	AtTRPV5	XP_019864931.1
	AtTRPL	XP_019869595.1
	AtTRPP	XP_019873676.1
	AtTRPML	XP_019870651.1
Dendroctonus ponderosae	*DpTRPA1*	XP_019770909.1
	DpPainless	XP_019757885.1
	DpPyrexia	XP_019767304.1
	DpTRPM	XP_019755240.1
	DpTRPV5	XP_019754025.1
	DpTRPV4	XP_019763585.1
	DpTRPML	XP_019754608.1
	DpTRPP	XP_019753887.1
	DpTRPL	XP_019770947.1
Aedes aegypti	*AaTRPA1*	XP_021698453.1
	AaPyrexia	XP_001648607.1
	AaPainless	XP_001649039.2
	AaTRPM	XP_021698945.1
	AaTRPV5	XP_001652424.2
	AaTRPL	XP_001651106.2
	AaTRPML	XP_021695946.1
Drosophila melanogaster	*DmTRPA1*	NP_001261602.1
	DmPainless	NP_611979.1
	DmInactive	NP_572353.1
	DmTRPL	NP_476895.1
	DmTRPML	NP_649145.1
	DmTRPN	NP_001303309.1
	DmWtrw	NP_731194.1
	DmNanchung	NP_648696.2
	DmTRPM	NP_001137672.2
	DmTRPgamma	NP_609802.1
	DmPyrexia	NP_612015.1
	DmTRPP	NP_609561.2
	DmTRP	NP_476768.1
